# Extract from Elkoplasty, or Anaplasty Applied to the Treatment of Old Ulcers

**Published:** 1855-07

**Authors:** Frank H. Hamilton

**Affiliations:** Surgeon to the Buffalo Hospital of the Sisters of Charity


### RECORD OF MEDICAL SCIENCE.

*Extract from Elkoplasty, or Anaplasty applied to the treatment of old Ulcers.* (A reply to Dr. Watson's "Reclamation.") By FRANK H. HAMILTON, A. M., M. D., Surgeon to the Buffalo Hospital of the Sisters of Charity.

Whether any cases ever occur in which an ulcer refuses to heal, chiefly or solely because of the great amount of integument, which has been lost, I will now consider.

I have said that ulcers generally close over by either the formation of new skin, or by the condensation of the granulations and the consequent contraction of the old skin. The limit to the centripetal contraction of the old skin is determined by its extensibility, qualified sometimes by the proximity of a joint, and the more or less flexure of the parts over which the integument is drawn. To this contraction there is, then, doubtless, a measure. It is not competent to the supply of every supposable loss of integument, and what hereafter remains to be done must be accomplished solely by the formation of new skin, and this mostly, if not wholly, as an outgrowth from the old. The exceptions which occur to this rule are too few to encourage us ever to rely upon them for aid. Let us determine, then, whether also to the formation of this new tissue there is a limit, or whether, on the contrary, it may continue to develop itself indefinitely. It will be fortunate if the latter supposition proves to be the correct one.

When an ulcer is healing, what do we observe? First, the granulations rise gradually towards the surface, until, along the margins at least, the level of the adjacent skin has been attained. At this moment, the new skin begins to form upon the peripheral granulations, as a pale pinkish circle, which becomes daily more and more opaque and white, until, in the end, it approaches very nearly to the color and firmness of the original structure. This process is at first rapid, and promises soon to close over the most extensive wounds in a brief period. And whether the wound is large or small, the same thing may be seen—the formation of new skin tissue is at first rapid, if the health and other circumstances [illegible] favorable. Soon, however, it is observed to progress more slo[illegible] ulcers, which were from the beginning quite small, we [illegible]prised to find how tedious, after a few days of rapid progress, are the final stages; and how long a time it takes to close up the last few lines of its surface. Nor is this change in the rapidity of the process by any means owing to a change in the health of the patient. It may often be observed to occur, and with the same uniformity of decline, when the health of the person affected is daily improving. Nor is it, again, because the extensibility of the skin in the vicinity has been exhausted, that this delay occurs, for I am speaking only of the new growth, and it is of this that I observe so constantly that *its* progress or development is daily more and more tardy. I wish to explain this phenomenon.

I think new skin tissue is formed, not simply upon, but in some sense *by* the old tissue. When, therefore, I say:—"The formative power of the old skin does not extend beyond a few lines. The new vessels, becoming more and more attenuated as they stretch inward from the periphery, lose, at length, the power of generating epithelial cells, or, if formed, they are too imperfectly organized to sustain an existence and they crumble away from the slightest provocation" (*page* 11 *of Article on Elkoplasty.*) I have sought only to express my convictions that the new tissue was dependent upon the old for its material of repair. A belief which, with modifications, is entertained by many pathologists in common with myself. Mr. Miller observes "As a general rule, integument is formed *by* and from integument,"* which expression he afterwards explains to mean, "It is not intended to be understood that the original skin sustains both the production of the organizable material, and the management of the organizing process; the major part of the blastema, whence the cuticular formation is produced, is doubtless furnished by the parts immediately beneath—granulations; and these may also contribute much to the organization. But the process of organization is *commenced* by the original skin, in that portion of the blastema with which it is in immediate contact; and continuance of the process is then, doubtless, maintained by those parts, whether recent or old, with which the advancing pellicle comes in contact."†

But certainly this is a point in pathology which is yet far from being absolutely determined. Whether the lymph which is to develop subsequently into epithelial cells has issued from the capillary vessels of the old skin, or from the vessels of the granulations, the microscope does not decide. The vessels from both of these structures are lying in close juxtaposition, if they are not actually in inosculation, wherever the epithelial cell or its plasma is sought to be obtained. The precise source, therefore, of this reparative material, remains at present a matter of conjecture. Mr. Miller believes that the old tissue furnishes the first plasma, and that subsequently the granulations assume this function entirely, leaving the old tissue no other duty, perhaps, than that of "management," or the assimilation of the products.

I wish to express my dissent from only the latter part of this hypothesis, and to explain more fully why I suppose that the old tissue continues to the last, not only the management, or adaptation of the new material, but also the production or effusion of the same; from which, also, I shall deduce the inference, that, when the old skin or its

* Principles of Surgery, by James Miller. 3d Amer. Ed., p. 199.
† Page 199.

vessels fail to furnish a supply of blastema, the cicatrization must generally cease.

*First.*—If the granulations or their appropriate vessels are competent, after the process has been commenced by the vessels of the old skin, to the formation of suitable plasma for the construction of epithelium—if this is with them a usual or a normal function, then ought they to perform this duty with as much speed and certainty as did the vessels of the old skin, whose function they have now assumed. But the fact is otherwise. The process, at the moment when it is believed that the skin has relinquished its productive agency to the granulations, languishes and finally ceases altogether.

If, however, we regard the new skin tissue, or the lymph of which it is to be constructed, as always a product of the old, or at least that the new material shall always require some element of matter from the old skin, which shall determine its development into epithelium, then we can readily understand how the gradual diminution of the capillaries through a new and always less perfectly organized structure shall at length render it impossible for them to furnish the plasma requisite for the repair, or for the construction of epithelium cells.

*Second.*—There are many analogies in the various processes of reproduction and repair which furnish support to this doctrine. Each tissue, when inflamed, furnishes a reparative material like that from which itself has been constructed. "When it is seen that in inflammations of bone the lymph usually ossifies; in those of ligament, is converted into a tough ligamentous tissue; and that, in general, lymph is organized into a tissue more or less corresponding with that from whose vessels it was derived; it is usually concluded that this happens under what is called the assimilative influence of the tissues adjacent to the organized lymph. But we may better explain the facts, by believing that the material formed in the inflammation of each part partakes, from the first, in the properties of the material products of that part; in properties which we know often determine the mode of formation independently of any assimilative force."*

I think, moreover, this case itself—the ulcer which continues to cicatrize more and more slowly—furnishes evidence that it is not by the law of analogous formations, or by assimilation, that epithelial cells are here determined, since this law would continue to operate to the consummation of the cure, and that without delay or abatement.

*Third.*—I have made a careful vertical section into the new skin, parallel to and about one line from the visible margin of a healing ulcer, and I have observed that the process of cicatrization, along this margin, was on the third or fourth day thereafter sensibly delayed. This I have repeated many times on ulcers, at various stages of cure, and always with the same result, provided the walls of the incision were kept only slightly asunder after the wound had been made.

I do not see how this incision should have disturbed the plastic or assimilative force of the free and yet unbroken margin, nor how the

* Lectures on Surgical Pathology, by James Paget. Amer. Ed., p. 223.

circumstance of retardation can be explained, if we believe the granulations below alone furnish the supplies for the new skin. And yet the fact finds a ready solution in the hypothesis that the old skin, or its capillaries, were the vehicles of nutriment for the new, and that by this bisection of their channels the supply has been cut off or interrupted.

My argument is, finally, that such cases as I have supposed, in which the ulcer will refuse to heal, solely because of the extent of tegumentary loss, do and must continue to occur, from the very laws which govern the cicatrization of such wounds.

That they do occur, I affirm also from my own observation and from the concurrent observation of others. Again and again do we find it necessary to amputate a limb, because the integument has been stripped from it beyond the power of nature to repair; and if the same results do not follow after extensive burns, it is, doubtless, because the original skin is only partially destroyed over all this surface, and there remains, therefore, a multitude of little islets from which new skin is capable of advancing.

As pertinent to this question, I will quote from a recent American reprint: "Cicatrization advances with greatest rapidity around the edges of the sore; the centre taking the longest time to heal, in consequence of the activity of the process appearing to diminish, the further the new skin extends from the old tissues. *Indeed, if the ulcer be large, there may not be sufficient for the cicatrization of the centre.*"*

But if it were true that all ulcers may in time be cured, under proper medical treatment, may it not be well to interfere in some cases of very much delayed healing? One of Dr. Watson's patients had waited ten years, and yet the sore refused to heal, and during a part of this time he was under Dr. Watson's care, and in bed. That all sores will not heal in ten years, even under good management, this case sufficiently illustrates; and I think there are very few large hospitals in which many similar cases might not be found.—*N. Y. Journal of Medicine.*

* Science and Art of Surgery, by John Erichsen. American Edition, p. 69.

